# Blame, PTSD and DSM-5: an urgent need for clarification

**DOI:** 10.1080/20008198.2018.1468709

**Published:** 2018-05-03

**Authors:** Talya Greene

**Affiliations:** Department of Community Mental Health, University of Haifa, Haifa, Israel

**Keywords:** DSM-5, blame, PTSD, diagnostic criteria, attributions, PCL-5, PSSI-5, DSM-5, culpa, TEPT, criterios diagnósticos, atribuciones, PCL-5, PSSI-5, DSM-5, 指责, 创伤后应激障碍, 诊断标准, 归因, PCL-5, PSSI-5, • There are fundamental problems with the new DSM-5 diagnostic criterion for PTSD: persistent distorted blame.• There is conflation of self-blame and other-blame which are two distinct phenomena heterogeneity in the research findings.• Distortion of blame may be complex to determine.• Standard assessment tools fail to accurately represent the criteria as currently formulated.• Clarification of PTSD diagnostic criteria is urgently needed.

## Abstract

DSM-5 substantially revised the PTSD criteria relating to exposure, redrawing symptom clusters and introducing additional symptom criteria, among them a newly defined criterion of persistent distorted blame of self or others. This commentary argues that there are fundamental problems with the current DSM-5 formulation of the blame criterion for PTSD. Most critically, there is conflation of self-blame and other-blame, which are two distinct phenomena, and there is heterogeneity in the research findings regarding the association between both kinds of blame and PTSD. Secondly, distortion of blame may be complex to determine. Finally, standard assessment tools fail to accurately represent the criteria as currently formulated. Despite the conceptual ambiguity in the diagnostic criteria and the lack of clarity regarding the assessment of this item in commonly-used measures, there is also evidence that blame is associated with other PTSD symptoms, is clinically relevant and may be an important intervention target in therapy. It is crucial, therefore, to clarify the blame criterion, differentiating aspects of self-blame and other-blame and, even more importantly, delineating the boundaries between normal and pathological blame.

## Abstract (es)

El DSM-5 revisó sustancialmente los criterios de TEPT relacionados con la exposición, reestableciendo los grupos de síntomas e introduciendo criterios de síntomas adicionales, entre ellos un criterio recientemente definido de culpa persistente y distorsionada de sí mismo o de los demás. Este comentario argumenta que hay problemas esenciales con la formulación actual del DSM-5 del criterio de culpa para el TEPT. De forma más crítica, hay una combinación de auto-culpa y culpa hacia los demás, que son dos fenómenos distintos, y hay heterogeneidad en los resultados de la investigación con respecto a la asociación entre ambos tipos de culpa y el TEPT. En segundo lugar, la distorsión de la culpa puede ser compleja de determinar. Finalmente, las herramientas estándar de evaluación no representan con precisión los criterios tal y como están formulados actualmente. A pesar de la ambigüedad conceptual en los criterios diagnósticos y la falta de claridad con respecto a la evaluación de este ítem en medidas comúnmente utilizadas, también hay evidencia de que la culpa está asociada con otros síntomas de PTSD, que es clínicamente relevante y quizá un objetivo importante de intervención en terapia. Por tanto, es crucial aclarar el criterio de la culpa, diferenciar los aspectos de la auto-culpa y la culpa de los demás, y aún más importante, delinear los límites entre la culpa normal y la patológica.

## Abstract (zh)

DSM-5大幅修订了与暴露相关的PTSD标准，重新绘制症状集群，并引入了其他症状标准，其中包括一个新定义的持续扭曲指责自我或他人的标准。本评论认为，目前DSM-5制定的PTSD指责标准存在根本性问题。最关键的是，自责和其他责任是两种截然不同的现象，关于这两种责任与PTSD之间的关联的研究结果中存在异质性。其次，指责的扭曲性可能很难确定。最后，标准评估工具无法准确地代表目前制定的标准。尽管诊断标准中存在概念上的模糊性，并且在常用测量中对该项目的评估缺乏清晰度，但也有证据表明指责与其他PTSD症状相关，并具有临床相关性，可能是治疗中重要的干预目标。因此，澄清责怪标准，区分自责和其他指责是至关重要的，甚至更重要的是划定正常和病理指责之间的界限。

DSM-5 made significant revisions to the posttraumatic stress disorder (PTSD) diagnostic construct including redefining the exposure criteria, redrawing symptom clusters and introducing additional symptom criteria (APA, 2013; Friedman, Resick, Bryant, & Brewin, 2011; Weathers, 2017). These changes have garnered some empirical support for their reliability (Freedman et al., 2013; Regier et al., 2013). Yet the revisions have been sharply criticized (Brewin, 2013; Galatzer-Levy & Bryant, 2013; Hoge et al., 2016; Miller, Wolf, & Keane, 2014), with questions raised about specificity, clinical utility and heterogeneity.

The revisions to the PTSD construct in DSM-5 include the addition of a symptom in the newly-defined negative alterations in mood and cognitions (NACM) cluster of ‘persistent distorted blame of self or others about the cause or consequences of the traumatic event(s)’ (criteria D3). Blame of self or others is a common reaction to traumatic events and, in some cases, may be normative, justified, appropriate and possibly helpful (Gray, Nash, & Litz, 2017). Yet, blame has been found to be associated with higher levels of PTSD in various studies (Cox, Resnick, & Kilpatrick, 2014).

This new ‘distorted blame’ criterion has not yet been well-studied, other than as part of a general exploration of the underlying dimensional structure of PTSD. Studies indicate that the D3 blame criteria loads well onto the new NACM cluster (Contractor et al., 2014; Elhai et al., 2012; Miller et al., 2013), while other studies suggest that blame may be part of a more narrow negative affect cluster which is differentiated from anhedonia symptoms (Armour et al., 2015; Liu et al., 2014). These studies do not address fundamental problems with the blame criterion as currently formulated.

The first critical issue is the conflation of two different phenomena – self-blame and other-blame – each of which has different associations and implications. Self-blame is a cognitive appraisal in which there is an internal attribution of responsibility for a negative event. This may be related to feelings of worthlessness and psychological distress (Zahn et al., 2015). Blame of others, conversely, reflects an external attribution of responsibility for the event, which could serve a self-protective function, reducing the need to make these negative internal attributions (Zinzow, Seth, Jackson, Niehaus, & Fitzgerald, 2010).

Research has indicated heterogeneous findings in the associations between both kinds of blame and PTSD. While some studies found self-blame to be associated with greater levels of PTSD (Cantón-Cortés, Cantón, & Cortés, 2012; Hassija & Gray, 2012; Moor & Farchi, 2011), others found that self-blame was associated with lower PTSD symptoms (Startup, Makgekgenene, & Webster, 2007) or was not associated with PTSD (DePrince, Chu, & Pineda, 2011). The findings related to other-blame are also mixed; some studies have indicated that other-blame is an effective coping strategy (Larsen & Fitzgerald, 2011), while others found that other-blame was associated with higher PTSD (Nickerson, Aderka, Bryant, & Hofmann, 2013; Zinzow et al., 2010). These inconsistent findings may be because the association between both self- and other-blame and PTSD might depend on the nature of the traumatic event (Reich et al., 2015) and cultural context (Wong & Tsai, 2007).

A second issue refers to the issue of ‘distortion’ of blame. Traumatic situations are often complex and multi-causal, making it hard for trauma survivors and mental health professionals to judge whether the blame has become ‘distorted’. It is also questionable whether the blame even needs to be ‘distorted’ in order to constitute an element of the PTSD construct; Delahanty et al. (1997), for example, found that when motor vehicle accident (MVA) survivors were indeed responsible for the accident, higher self-blame was associated with more distress.

Finally, there is a lack of consistency between the DSM-5 criteria and their application via standard assessment tools, particularly self-report measures. The PCL-5 (Weathers et al., 2013) formulates this item as: ‘blaming yourself or someone else for the stressful experience or what happened after it’, omitting the distortion aspect. The PSSI-5 (Foa et al., 2016) clarifies that a person may make comments like ‘I should have known’. Yet blame (of self or others) may actually be an understandable and possibly helpful reaction to an event as the survivor attempts to understand and process their experiences, and perhaps take responsibility where appropriate (Gray et al., 2017). Applying the standard assessment tools in their current form, however, may run the risk of reframing this understandable coping response as psychopathology.

Does this mean we should exclude blame from the DSM-5 criteria? There is not yet a clear answer to this question. Despite the conceptual ambiguity in the diagnostic criteria, the heterogeneity in the research findings and the lack of clarity regarding the assessment of this item in commonly-used measures, there is also evidence that blame is associated with other PTSD symptoms, is clinically relevant, might help distinguish PTSD from other disorders, could provide information about the traumatic event itself and may be an important intervention target in therapy (Cox et al., 2014; Friedman et al., 2011; Taylor, 2017).

It is crucial, therefore, to clarify the blame criterion, differentiating aspects of self-blame and other-blame and, even more importantly, delineating the boundaries between normal and pathological blame. Future research could then more sensitively and specifically assess whether blame ought to be part of the PTSD construct, and whether it matters to whom blame is attributed or if the blame attribution is distorted. As diagnostic criteria are formulated and reformulated, and amidst the often-valid criticism regarding DSM-5, there is an urgent need for clarification.
